# Genome-Wide Association Study Identifies Candidate Genes for Starch Content Regulation in Maize Kernels

**DOI:** 10.3389/fpls.2016.01046

**Published:** 2016-07-27

**Authors:** Na Liu, Yadong Xue, Zhanyong Guo, Weihua Li, Jihua Tang

**Affiliations:** ^1^College of Biological Engineering, Henan University of TechnologyZhengzhou, China; ^2^State Key Laboratory of Wheat and Maize Crop Science/Collaborative Innovation Center of Henan Grain Crops, Henan Agricultural UniversityZhengzhou, China; ^3^Hubei Collaborative Innovation Center for Grain Industry, Yangtze UniversityJinzhou, China

**Keywords:** GWAS, starch synthesis, gene ontology analysis, maize (*Zea mays* L.), ADP-glucose pyrophosphorylase

## Abstract

Kernel starch content is an important trait in maize (*Zea mays* L.) as it accounts for 65–75% of the dry kernel weight and positively correlates with seed yield. A number of starch synthesis-related genes have been identified in maize in recent years. However, many loci underlying variation in starch content among maize inbred lines still remain to be identified. The current study is a genome-wide association study that used a set of 263 maize inbred lines. In this panel, the average kernel starch content was 66.99%, ranging from 60.60 to 71.58% over the three study years. These inbred lines were genotyped with the SNP50 BeadChip maize array, which is comprised of 56,110 evenly spaced, random SNPs. Population structure was controlled by a mixed linear model (MLM) as implemented in the software package TASSEL. After the statistical analyses, four SNPs were identified as significantly associated with starch content (*P* ≤ 0.0001), among which one each are located on chromosomes 1 and 5 and two are on chromosome 2. Furthermore, 77 candidate genes associated with starch synthesis were found within the 100-kb intervals containing these four QTLs, and four highly associated genes were within 20-kb intervals of the associated SNPs. Among the four genes, *Glucose-1-phosphate adenylyltransferase* (*APS1*; Gene ID GRMZM2G163437) is known as an important regulator of kernel starch content. The identified SNPs, QTLs, and candidate genes may not only be readily used for germplasm improvement by marker-assisted selection in breeding, but can also elucidate the genetic basis of starch content. Further studies on these identified candidate genes may help determine the molecular mechanisms regulating kernel starch content in maize and other important cereal crops.

## Introduction

Maize (*Zea mays* L.) is one of the most widely grown crops in the world, serving as the staple food for more than 900 million people. Maize seeds (i.e., kernels) provide a rich source of carbohydrates, mostly in the form of starch, which accounts for 65–75% of kernel dry weight. Therefore, the seed yield of maize is largely determined by the efficient biosynthesis and storage of starch (Boyer and Hannah, 2001). Accordingly, improving the kernel starch content could yield higher value products in the corn processing industry (Goldman et al., 1993).

Starch biosynthesis is a complicated process. In brief, sucrose is transported to the sink (e.g., kernels) and converted to uridine diphosphate (UDP)-glucose and fructose by sucrose synthase encoded by the *Sh1* gene (Carlson and Chourey, 1996), and then UDP-glucose serves as the primary precursor for starch biosynthesis. In the endosperm, starch biosynthesis is coordinated by a series of enzymes, including ADP-glucose pyrophosphorylase (AGPase), soluble starch synthase (SS), starch branching enzyme (SBE), starch debranching enzyme (SDBE), and plastidial starch phosphorylase (Pho1), whereas amylose is synthesized by AGPase and granule-bound starch synthase (GBSS; Jeon et al., 2010). AGPase, mainly present in maize endosperm, embryo, and leaf tissue, is a heterotetramer comprised of two structurally related dimerized polypeptides designated as the large subunits (LS) and the small subunits (SS), which are encoded by the *Shrunken2* (*Sh2*) and *Brittle2* (*Bt2*) genes, respectively (Huang et al., 2014). Overexpression of related genes, such as *Bt2*, *Sh2*, and *Shrunken1* (*Sh1*), can induce significant increases of the starch content in maize endosperm (Hannah et al., 2012; Jiang et al., 2013). In contrast, mutations occurred at the starch accumulation genes can usually cause starch content decreases. For example, the null mutant of AGPase LS maize endosperm results in 25–30% less starch content relative to the wild type (Tsai and Nelson, 1966). Additionally, the two transcripts encoding AGPase LS, namely *agpsemzm* and *agpllzm*, contributed to approximately 7% of the total endosperm starch content (Huang et al., 2014).

Kernel starch content is a typical quantitative trait controlled by a large number of quantitative trait loci (QTL). Despite recent progress in understanding starch biosynthesis and accumulation at molecular levels, QTLs underlying phenotypic variation in starch content across maize lines are still to be defined (Willmot et al., 2006; Dudley et al., 2007; Chen et al., 2012). Additionally, inconsistencies in the number and locations of the detected QTLs for kernel starch content have been reported using traditional QTL markers (Li et al., 2009; Wang et al., 2010; Yang et al., 2013). These inconsistent results are probably caused by the limited population size and genetic diversity within mapping populations, and the resultant type I or type II errors are often further magnified by the use of traditional molecular markers, which can only capture a small portion of the genetic diversity of the species. Genome-wide association studies (GWAS) with SNP arrays are an alternative to the traditional QTL-mapping approaches using markers such as SSRs, AFLPs, RFLPs, and SRAPs. Compared with traditional QTL mapping approaches, association mapping has exhibited several advantages in exploring the genetic mechanism underlying the complex agronomic traits. First, the populations used in association mapping can be collections of individual varieties, inbred lines, or landraces with varying traits. Diversified historical recombination events among different lineages make it possible to discover linked markers associated with causative genes (Xue et al., 2013; Wen et al., 2014). Second, the application of SNP arrays allows the detection of 10s of 1000s of loci simultaneously. Mutations or standing variation may influence the expression and functionality of genes and ultimately lead to different agronomically important characters. The main objectives of this study were to identify QTLs controlling kernel starch content in maize through GWAS using newly developed robust maize SNP50 array (Ganal et al., 2011) and to predict the possible candidate genes responsible for starch synthesis and accumulation.

## Materials and Methods

### Plant Material

Accurate quantification of the target trait is a prerequisite for molecular mapping and a biased population structure can lead to false positive associations (i.e., type I error). In this study, a population comprised of a global core collection of 263 maize inbred lines was used to represent a wide range of diversity, among them, 71 inbred lines are from tropical and subtropical zones, while the remainder are from temperate regions. In order to ensure the normal flowering and pollination of the tropical germplasm, we choose to conduct this study in the tropical regions of Sanya. All the inbred lines were planted in a field in Ledong (Hainan Province, 18.75°N, 109.17°E) in Nov. 2011, 2012, and 2013. The average annual rainfall total was 1,181 mm. The average annual temperature is 23°C. The average annual sunshine total is 1039.6 h. The accumulated temperature (in excess of 10°C) is 9,300.7°C. In the field plot, 24 seeds of each maize inbred line were planted in a randomized complete block design with three replicates (across growing seasons). In one block, eight seeds of each maize inbred line were planted in a row, leaving a 20-cm gap between each line. During the growing seasons, plants were irrigated and given extra doses of pollen by hand. The mature seeds of each inbred line were harvested in bulk and used for starch content analysis.

### Determination of Starch Content

For three consecutive years, the maize kernels of 263 lines were harvested and analyzed each year to minimize variation across different years. The subsampled kernels from the bulk harvest were used for starch content measurement with a near-infrared analyzer (model: MATRIX-1, Bruker Corporation, Karlsruhe, Germany) according to the method described by Dudley and Lambert (1992). The uniform kernels of maize were filled into the sample cup, and the excess corn was scraped off with a ruler. Every sample from every year was analyzed three times to generate technical replicates, and the average values of the three replicates were calculated for further analyses. The data was analyzed using the ANOVA method as implemented in SPSS 18.0 (IBM Corp., Armonk, NY, USA). Broad-sense heritability was also calculated according to the method developed by Knapp et al. (1985) with the formula *H*^2^*_B_* (%) = σ^2^*_G_*/(σ^2^*_G_* + σ^2^*_GE_/n* + σ^2^*_e_/nb*) ^∗^100, where σ^2^*_G_* is the genetic variance, σ^2^*_GE_* is the genotype × environment interaction variance, σ^2^*_e_* is the error variance, *n* is the number of environments, and *b* is the number of replications in each experiment. A frequency map was made using Origin 8.0 (OriginLab Corporation, Northampton, MA, USA). Using an R package, the best linear unbiased prediction (BLUP) values were estimated from the average value of each inbred line across the 3 years. The BLUP values were used as the input data for the association mapping analysis.

### Association Mapping Analysis

A modified CTAB procedure was followed for DNA extraction in this study (Healey et al., 2014), and the DNA quality and concentration were verified by gel-electrophoresis and spectrophotometer before genotyping.

The panel consisting of 263 inbred lines was genotyped with the Illumina MaizeSNP50 chip (Illumina, Inc., San Diego, CA, USA) according to the method described by Ganal et al. (2011), which was also applied in previous studies (Xue et al., 2013). The kinship matrix was calculated using the Loiselle algorithm (Loiselle et al., 1995) to determine relatedness among the sampled individuals. With the two matrixes, a mixed linear model (MLM) was applied to analyze the dataset using the GWAS software GAPIT (Genome Association and Prediction Integrated Tool-R package; Lipka et al., 2012), and principal components (PCs) were used to control for population structure (quantile–quantile plots showed over fitting of a PC + K model). These methods were used for genome-wide association mapping with 52,370 SNPs (MAF > 0.05).

### Gene Ontology Analysis

The available genome sequence of the maize line B73 was used as the reference genome for candidate gene analyses (Schnable et al., 2009). The approximately 120-bp SNP probe sequences (Illumina, Inc.) were used as queries to BLAST against MaizeGDB (Lawrence et al., 2004)^1^. A 100-kb window was selected in order to fall within the estimated 100-kb window of linkage disequilibrium (LD) decay that occurs in our association panel. The genes within this window size were identified in MaizeGDB according to the positions of the closest flanking significant SNPs (*P* < 0.0001) or supporting intervals (Maize genetics and genomics database, 2015)^2^. The functions of corresponding genes were predicted using the Blast2Go program (Conesa et al., 2005).

## Results

### Variation in Starch Content among the Maize Inbred Lines

**FIGURE 1:**
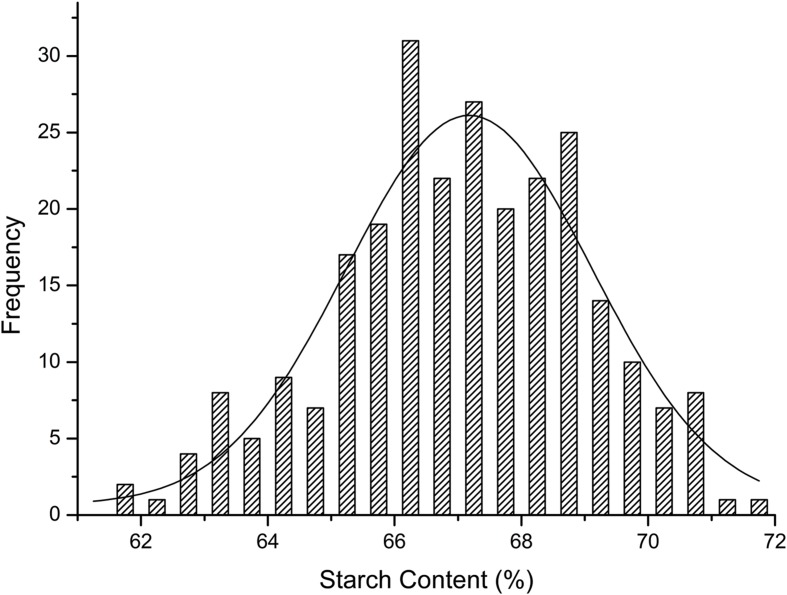
**Frequency map of starch content**.

The starch contents of the 263 maize lines (**Supplementary Table S1**) were measured using the NIRS method. In this panel, the average kernel starch content was 66.99% with a range from 60.60% to 71.58% (**Table 1**). Additionally, the starch content showed a high heritability of 88.30%. The majority of inbred lines produced kernels that were of 65–70% starch content exhibited a normal distribution (*r*^2^ = 0.88; **Figure 1**). Starch content varied significantly among different germplasms (*P* ≤ 0.05; **Table 1**). These results suggested that the selected population was suitable for the association analysis of kernel starch content.

**Table 1:** ANOVA of starch contents among maize inbred lines.

Trait	Mean ±*SE*	Range	95% confidence level		*H^2^_B_* (%)
				
			Lower limit	Upper limit	
Starch content	66.99 ± 0.13	60.60–71.58	66.18	71.58	88.3

	**SS**	**DF**	**MF**	***F***	***P***

Between groups	10122.179	262	38.634	22.172	0
Within group	3666.162	2104	1.742		
Total	13788.341	2366			


### Linkage Disequilibrium in the Association Panel

**FIGURE 2:**
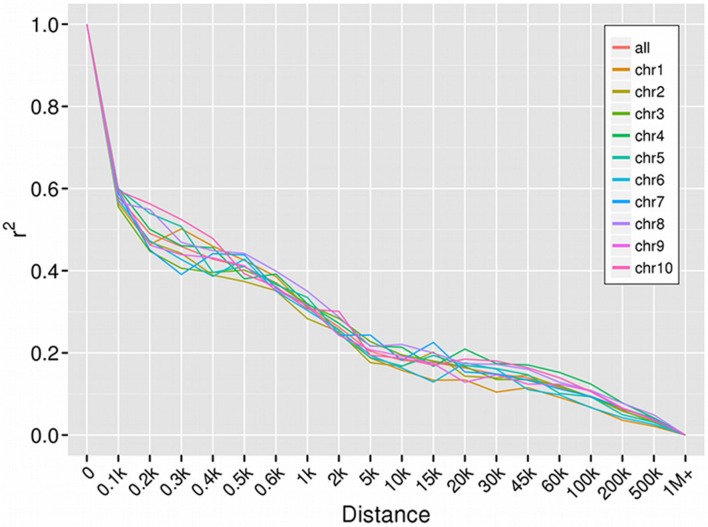
**Linkage disequilibrium decay of each chromosome**.

All 52,370 SNPs (MAF > 0.05) were used as input data to calculate the genome-wide LD in the present panel. A rapid decline in LD was observed with increasing physical distance on all chromosomes (Chr), but the decay rate varied among Chr (**Figure 2**). LD was reached within 30–45 kb on Chr 1, 50–60 kb on Chr 4 and 5, and 80–150 kb on the remaining Chr. The mean length of LD decay across all Chr was 80–100 kb (*r*^2^ = 0.1). At a cut-off of *r*^2^ = 0.2, the mean length of LD decay decreased rapidly to 5 kb.

### Genome-Wide Association Study and SNP Discovery

**FIGURE 3:**
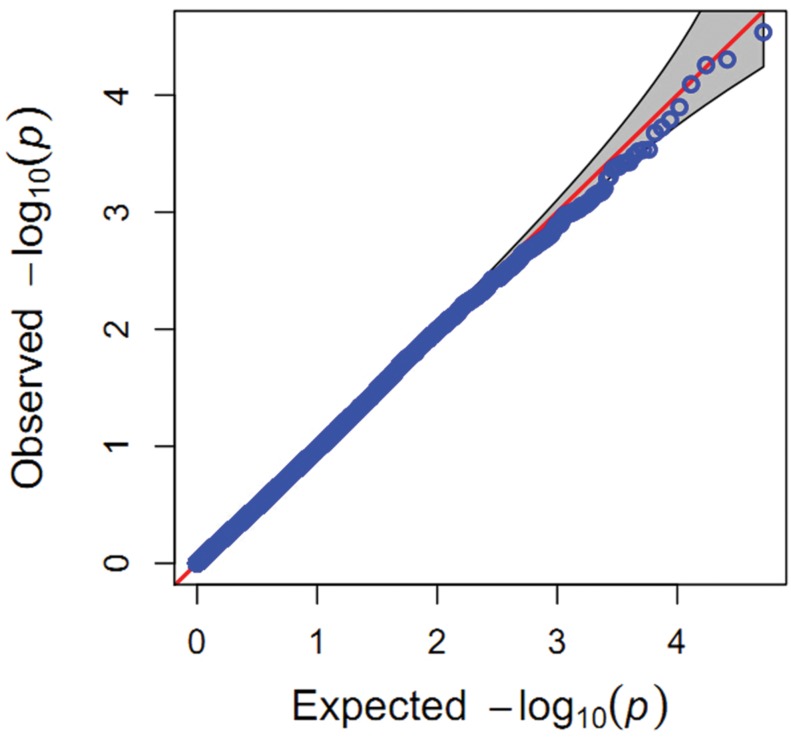
**Quantile–quantile plot for associations with starch content.** *P*-values are shown on a -log_10_ scale, and the dashed horizontal line indicates the genome-wide significance threshold (-log *P* ≥ 3).

**FIGURE 4:**
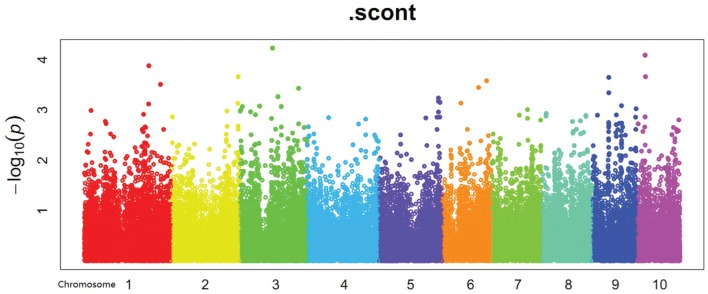
**Manhattan plot of starch content abscissa means for maize chromosomes 1–10**.

In the present study, population structure was controlled for by using the PC matrix, with six PC axes explaining about 6.9% of the variance. Quantile–quantile plots (**Figure 3**) showed that the MLM model with six PC axes effectively accounted for the population substructure of starch content. A total of four SNPs (**Supplementary Table S2**) significantly associated (*P* < 0.0001) with starch content were detected on Chr 1, 2, and 5 (**Figure 4**), among which SNP SYN1878 was the most significant (*P* = 2.9 × 10^-5^). These results are consistent with the quantitative nature of starch content, which is known to be controlled by a large number of genes with small effects.

The overall LD decay across the genome of this panel was 100 kb; as such, a 200-kb region flanking the left and right side of each SNP was defined as a QTL, which were then used to identify genes from the maize genome browser publically available at www.maizegdb.org. A total of 77 candidate genes were found in these associated regions (listed in **Supplementary Table S2**). The genes within this 200-kb window size were identified through MaizeGDB according to the positions of the closest flanking SNPs or supporting intervals^3^. Based on the gene annotation of the B73 maize genome, some genes that are likely involved in starch synthesis were subjected to gene ontology (GO) analysis. The Blast2Go program was used to predict the functions of these genes (**Table 2**), and at least two of these genes or their orthologs were known functional genes involved in the starch synthesis pathway: (GRMZM2G163437, which encodes the ADP glucose pyrophosphorylase SS, and GRMZM2G450163, which encodes 6-phospho-fructokinase).

**Table 2:** Candidate gene functions by Blast2Go.

Gene ID	Sequence description	Sequence length (bp)	Hits	Minimum eValue	GOs	GO terms
GRMZM2G163437	adp-glucose pyrophosphorylase small subunit	4,593	8	1.1E-121	10	cytosol^C^; heterotetrameric ADPG pyrophosphorylase complex^C^; amyloplast^C^; photoperiodism^P^; ATP binding^F^; starch biosynthetic process^P^; chloroplast stroma^C^; glucose-1-phosphate adenylyltransferase activity^F^; glycogen biosynthetic process^P^; apoplast^C^
GRMZM2G064179	transposon en spm sub-class	1,109	11	11	1	plastid^C^
GRMZM2G450163	6-phosphofructokinase	6687	No	No	5	glycolysis^P^; 6-phosphofructokinase activity^F^; ATP binding^F^; diphosphate-fructose-6-phosphate-1-phosphotransferase activity^F^; 6-phosphofructokinase complex^C^


*Gene ontology (GO) terms are categorized and denoted as cellular components (C), molecular functions (F), or biological processes (P) according to their superscripts.*

## Discussion

### NIRS for Kernel Starch Content Analysis

Traditional starch content measurement protocols employ kernel powdering and starch hydrolysis to measure starch content according to the percentage of hydrolyzed soluble sugar glucose in the dry kernel (Yemm and Willis, 1954; Li et al., 2011). This method is not only laborious and time-consuming, but also damages the integrity of maize kernels that could have value in breeding programs. On the contrary, NIRS is a fast, reliable, and non-destructive method for the evaluation of such breeding traits, especially using bulk materials (Plumier et al., 2013). The reliability of NIRS for measuring maize kernel starch content has been previously confirmed by Fang (2011), who found a remarkable positive correlation (*r*^2^ = 0.961) between the values measured by NIRS and those obtained by traditional chemical methods. In our study, the NIRS method also yielded reliable results as demonstrated by the small deviations (<5% in most cases) among the biological replicates (**Supplementary Table S1**).

### Population Structure for Association Mapping of Kernel Starch Content

In previous studies, kernel starch content was often studied together with protein or oil content (Wassom et al., 2008; Li et al., 2009; Yang et al., 2013) and most genetic materials used in these previous experiments were derived from high oil maize lines. To conduct a proper GWAS on kernel starch content in maize, it was necessary to test an unbiased population with a sufficient number of individuals from among diverse genetic backgrounds. In our study, the association panel was comprised of tropical, subtropical, and temperate lines from regions including South Asia, North and South America, Europe, and the Middle East, representing the core diversity of the broader species population that has been characterized previously (Yan et al., 2009; Xue et al., 2013). Our study particularly focused on results obtained in the Hainan seed base because the primary agronomic features of these lines have been preliminarily evaluated in various maize breeding programs. Owing to previously demonstrated significant genotype-by-environment effects (Visioni et al., 2013), the interaction between genotype and environmental conditions was considered in the design of this study, and starch content data from 3 years (2011, 2012, and 2013) was used to minimize the effect of growing environment on starch content.

### Maize Kernel Starch Content Is Genetically Controlled by Many Small Effect QTLs

The complex genetic architecture of starch content has been demonstrated through long-term selection experiments using inbred lines (Moose et al., 2004). The continued phenotypic response of kernel composition provides convincing evidence that starch content is controlled by a number of small effect QTLs as demonstrated by other studies using near isogenic lines (Goldman et al., 1993; Clark et al., 2006; Dudley, 2008; Wassom et al., 2008; Li et al., 2009; Cook et al., 2012). The advent of high-density DNA marker linkage maps in many plant species has provided an opportunity to identify QTLs (Liu et al., 2008; Sun et al., 2008; Li et al., 2009). Through this GWAS, we were able to detect substantially more reliable QTLs. The information provided in this study can serve as the starting point for functional gene studies to clarify the genetic mechanisms underlying starch content diversity among different maize lines.

### Potential Genes Involved in Kernel Starch Biosynthesis and Accumulation Revealed by GWAS

The maize SNP50 BeadChip maize array used in this study contains a total of 56,110 SNPs. Given the 2,300-Mb genome size of maize, there is approximately 50 kb of total sequence for each SNP marker used in this study. Therefore, a significantly associated SNP may represent an approximately 100-kb region covering the 50 kb upstream and downstream along each chromosome. Thanks to the publically available maize genome browser, we were able to browse and retrieve the coding gene information from the associated QTLs (Wang et al., 2013).

Efforts were made to utilize functionally characterized maize genes for starch content regulation. Four structural genes influence starch synthesis in maize: *Sh2*, *Bt2*, *opaque-2* (*O2*), and *Sh1* (Goldman et al., 1993). Some functional genes involved in metabolic reactions were also detected in this study. For example, through GO analysis, the *Glucose-1-phosphate adenylyltransferase* (*APS1*) gene (gene ID GRMZM2G163437) was identified as a closely linked gene; this protein presumably catalyzes the alpha-D-glucose-1-phosphate to ADP-glucose (ADPG) pathway and plays an important role in starch synthesis (Li et al., 2009). Another identified gene (gene ID GRMZM2G450163) encodes 6-phospho-fructokinase, which is involved in the Embden–Meyerhof Pathway (EMP). The EMP is important to the energy cycle as it converts fructose to glucose-1-phosphate. A transposable element (gene ID GRMZM2G128149) was also identified. As the maize genome is heavily duplicated and full of gene insertions owing to transposable elements, it is not surprising to see that this particular transposon might be involved in starch content regulation. Similarly, Liu et al. (2014) reported a transposable element insertion that disturbed the starch synthase gene *SSIIb* in maize and thus altered starch content.

## Future Perspectives

The GWAS presented here was able to uncover associations between SNPs and kernel starch content in maize. Although this methodology only provides a statistical link between traits and genomic sequences, such information can be a solid starting point for functional genetic studies. The gene candidates identified by our GWAS will be profoundly enhanced as additional molecular evidence (e.g., via transgenic approaches) becomes available. Furthermore, SNPs within candidate genes can also be used to further test the contributions of these genes to traits like kernel starch content.

## Author Contributions

This study was conceived by JT and NL. GWAS and GO analyses were conducted by YX and ZG. Collections of maize were performed by WL. NL wrote the manuscript. All authors read and approved the final manuscript.

## Conflict of Interest Statement

The authors declare that the research was conducted in the absence of any commercial or financial relationships that could be construed as a potential conflict of interest.

## Footnotes

1 http://archive.maizegdb.org/

2 http://gbrowse.maizegdb.org/gb2/gbrowse/maize_v2/

3 http://gbrowse.maizegdb.org/gb2/gbrowse/maize_v2/

## Supplementary Material

**TABLE S1 | Kernel starch contents of the 263 maize lines.**

**TABLE S2 | SNPs, QTLs, and candidate genes significantly associated (*P* < 0.0001) with starch content.**

## References

[B1] Boyer C. D. Hannah L. C. (2001). “Kernel mutants of corn,” in *Specialty Corns*, ed. Hallauer A. R. (Boca Raton, FL: CRC), 1–31.

[B2] Carlson S. J. Chourey P. S. (1996). Evidence for plasma membrane associated forms of sucrose synthase in maize. *Mol. Gen. Genet.* 252 303–310. 10.1007/BF02173776 8842150

[B3] Chen J. Zhang J. Liu H. Hu Y. Huang Y. (2012). Molecular strategies in manipulation of the starch synthesis pathway for improving storage starch content in plants (review and prospect for increasing storage starch synthesis). *Plant Physiol. Biochem.* 61 1–8. 10.1016/j.plaphy.2012.08.013 23023581

[B4] Clark D. Dudley J. W. Rocheford T. R. LeDeaux J. R. (2006). Genetic analysis of corn kernel chemical composition in the random mated 10 generation of the cross of generations 70 of IHO x ILO. *Crop Sci.* 46 807–819. 10.2135/cropsci2005.06-0153

[B5] Conesa A. Götz S. García-Gómez J. M. Terol J. Talón M. Robles M. (2005). Blast2GO: a universal tool for annotation, visualization and analysis in functional genomics research. *Bioinformatics* 21 3674–3676. 10.1093/bioinformatics/bti610 16081474

[B6] Cook P. McMullen M. D. Holland J. B. Tian F. Bradbury P. Ross-Ibarra J. (2012). Genetic architecture of maize kernel composition in the nested association mapping and inbred association panels. *Plant Physiol.* 158 824–834. 10.1104/pp.111.185033 22135431 PMC3271770

[B7] Dudley J. W. (2008). Epistatic interactions in crosses of Illinois high oil 3 Illinois low oil and of Illinois high protein 3 Illinois low protein corn strains. *Crop Sci.* 48 59–68. 10.2135/cropsci2007.04.0242

[B8] Dudley J. W. Clark D. Rocheford T. R. LeDeaux J. R. (2007). Genetic analysis of corn grain chemical composition in the random mated 7 generation of the cross of generations 70 of IHP x ILP. *Crop Sci.* 47 45–57. 10.2135/cropsci2006.03.0207

[B9] Dudley J. W. Lambert R. J. (1992). Ninety generations of selection for oil and protein in maize. *Maydica* 37 81–87.

[B10] Fang Y. (2011). Nondestructive analysis of crude starch in whole kernel maize by near infrared reflectance spectroscopy. *Crops* 2 25–27.

[B11] Ganal M. W. Durstewitz G. Polley A. Bérard A. Buckler E. S. Charcosset A. (2011). A large maize (*Zea mays* L.) SNP genotyping array: development and germplasm genotyping, and genetic mapping to compare with the B73 reference genome. *PLoS ONE* 6:e28334 10.1371/journal.pone.0028334 PMC3234264 22174790

[B12] Goldman I. L. Rocheford T. R. Dudley J. W. (1993). Quantitative trait loci influencing protein and starch concentration in the Illinois Long Term Selection maize strains. *Theor. Appl. Genet.* 87 217–224. 10.1007/BF00223767 24190215

[B13] Hannah L. C. Futch B. Bing J. Shaw J. R. Boehlein S. Stewart J. D. (2012). A shrunken-2 transgene increases maize yield by acting in maternal tissues to increase the frequency of seed development. *Plant Cell* 24 2352–2363. 10.1105/tpc.112.100602 22751213 PMC3406911

[B14] Healey A. Furtado A. Cooper T. Henry R. J. (2014). Protocol: a simple method for extracting next-generation sequencing quality genomic DNA from recalcitrant plant species. *Plant Methods* 10 21–28. 10.1186/1746-4811-10-21 25053969 PMC4105509

[B15] Huang B. Q. Hennen-Bierwagen T. A. Myers A. M. (2014). Functions of multiple genes encoding ADP-Glucose pyrophosphorylase subunits in maize endosperm, embryo, and leaf1. *Plant Physiol.* 164 596–611. 10.1104/pp.113.231605 24381067 PMC3912092

[B16] Jeon J. S. Ryoo N. Hahn T. R. Walia H. Nakamura Y. (2010). Starch biosynthesis in cereal endosperm. *Plant Physiol. Biochem.* 48 383–392. 10.1016/j.plaphy.2010.03.006 20400324

[B17] Jiang L. L. Yu X. M. Xin Q. X. Yu Q. Deng S. Bai B. (2013). Multigene engineering of starch biosynthesis in maize endosperm increases the total starch content and the proportion of amylase. *Transgenic Res.* 22 1133–1142. 10.1007/s11248-013-9717-4 23740205

[B18] Knapp S. J. Stroup O. B. Ross W. M. (1985). Exact confidence intervals for heritability on a progeny mean basis. *Crop Sci.* 25 192–194. 10.1007/BF00288995

[B19] Lawrence C. J. Dong Q. Polacco M. L. Seigfried T. E. Brendel V. (2004). MaizeGDB, the community database for maize genetics and genomics. *Nucleic Acids Res.* 32 D393–D397. 10.1093/nar/gkh011 14681441 PMC308746

[B20] Li N. Zhang S. J. Zhao Y. J. Li B. Zhang J. R. (2011). Over-expression of AGPase genes enhances seed weight and starch content in transgenic maize. *Planta* 233 241–250. 10.1007/s00425-010-1296-5 20978801

[B21] Li Y. Wang Y. Wei M. Li X. Fu J. (2009). QTL identification of grain protein concentration and its genetic correlation with starch concentration and grain weight using two population in maize (*Zea mays* L). *J. Genet.* 88 61–67. 10.1007/s12041-009-0008-z 19417545

[B22] Lipka A. E. Tian F. Wang Q. Peiffer J. Li M. Bradbury P. J. (2012). GAPIT: genome association and prediction integrated tool. *Bioinformatics* 28 2397–2399. 10.1093/bioinformatics/bts444 22796960

[B23] Liu H. H. Liu H. Q. Wei L. Yang X. H. Lin Z. W. (2014). A transposable element insertion disturbed starch synthase gene SSIIb in maize. *Mol. Breed.* 34 1159–1171. 10.1007/s11032-014-0107-2

[B24] Liu Z. H. Xie H. L. Tian G. W. Chen S. J. Wang C. L. Hu Y. M. (2008). QTL mapping of nutrient components in maize kernels under low nitrogen conditions. *Plant Breed.* 127 279–285. 10.1111/j.1439-0523.2007.01465.x

[B25] Loiselle B. A. Sork V. L. Nason J. Graham C. (1995). Spatial genetic structure of a tropical understory shrub, *Psychotria officinalis* (Rubiaceae). *Am. J. Bot.* 82 1420–1425. 10.2307/2445869

[B26] Maize genetics and genomics database (2015). *Maize Genetics and Genomics Database.* Available at: http://gbrowse.maizegdb.org/gb2/gbrowse/maize_v2/ (accessed June 2015).

[B27] Moose S. P. Dudley J. W. Rocheford T. R. (2004). Maize selection passes the century mark: a unique resource for 21st century genomics. *Trends Plant Sci.* 9 358–364. 10.1016/j.tplants.2004.05.005 15231281

[B28] Plumier B. Danao M. J. Singh V. Rausch K. (2013). “Analysis and prediction of unreacted starch content in corn using FT-NIR spectroscopy,” in *Proceedings of the Conference Paper in Transactions of the American Society of Agricultural and Biological Engineers Paper No 131596314*, Kansas City, MO.

[B29] Schnable P. S. Ware D. Fulton R. S. Stein J. C. Wei F. Pasternak S. (2009). The B73 maize genome:- complexity, diversity, and dynamics. *Science* 326 1112–1115. 10.1126/science.1178534 19965430

[B30] Sun H. Y. Lu J. H. Fan Y. D. Zhao Y. Kong F. M. Li R. J. (2008). Quantitative trait loci (QTLs) for quality traits related to protein and starch in wheat. *Prog. Nat. Sci.* 18 825–831. 10.1016/j.pnsc.2007.12.013

[B31] Tsai C. Y. Nelson O. E. (1966). Starch-deficient maize mutant lacking adenosine diphosphate glucose pyrophosphorylase activity. *Science* 151 341–343. 10.1126/science.151.3708.341 5903344

[B32] Visioni A. Tondelli A. Francia E. Alexander P. A. Malosetti M. Russell J. (2013). Genome-wide association mapping of frost tolerance in barley (*Hordeum vulgare* L.). *BMC Genomics* 14:424 10.1186/1471-2164-14-424 PMC3701572 23802597

[B33] Wang J. Kong L. Gao G. Luo J. (2013). A brief introduction to web-based genome browsers. *Brief. Bioinform.* 14 131–143. 10.1093/bib/bbs029 22764121

[B34] Wang Y. Z. Li J. Z. Li Y. L. Wei M. G. Li X. H. Fu J. F. (2010). QTL detection for grain oil and starch content and their associations in two connected F2:3 populations in high-oil maize. *Euphytica* 174 239–252.

[B35] Wassom J. J. Wong J. C. Martinez E. King J. J. DeBaene J. Hotchkiss J. R. (2008). QTL associated with maize kernel oil, protein, and starch concentrations; kernel mass; and grain yield in Illinois high oil x B73 backcross-derived lines. *Crop Sci.* 48 243–252. 10.2135/cropsci2007.04.0208

[B36] Wen Z. Tan R. Yuan J. Bales C. Du W. Zhang S. (2014). Genome-wide association mapping of quantitative resistance to sudden death syndrome in soybean. *BMC Genomics* 15:809 10.1186/1471-2164-15-809 PMC4189206 25249039

[B37] Willmot D. B. Dudley J. W. Rocheford T. R. Bari A. (2006). Effect of random mating on marker-QTL associations for grain quality traits in the cross of Illinois High Oil x Illinois Low Oil. *Maydica* 51 187–199.

[B38] Xue Y. D. Warburton M. L. Sawkins M. Zhang X. H. Setter T. Xu Y. B. (2013). Genome-wide association analysis for nine agronomic traits in maize under well-watered and water-stressed conditions. *Theor. Appl. Genet.* 126 2587–2596. 10.1007/s00122-013-2158-x 23884600

[B39] Yan J. Shah T. Warburton M. L. Buckler E. S. McMullen M. D. Crouch J. (2009). Genetic characterization and linkage disequilibrium estimation of a global maize collection Using SNP Markers. *PLoS ONE* 4:e8451 10.1371/journal.pone.0008451 PMC2795174 20041112

[B40] Yang G. Dong Y. Li Y. Wang Q. Shi Q. Zhang X. H. (2013). Verification of QTL for grain starch content and its genetic correlation with oil content using two connected RIL populations in high-oil maize. *PLoS ONE* 8:e53770 10.1371/journal.pone.005377 PMC3540064 23320103

[B41] Yemm E. W. Willis A. J. (1954). The estimation of carbohydrates in plant extracts by the anthrone. *Biochemistry* 57 508–514. 10.1042/bj0570508 PMC1269789 13181867

