# Supplementary material for: Genome-Wide Association Study Identifies Candidate Genes for Starch Content Regulation in Maize Kernels
Source: Front Plant Sci. 2016 Jul 27;7:1046. doi: 10.3389/fpls.2016.01046 (PMC4961707; doi:10.3389/fpls.2016.01046)
Supplement: Supplementary file 1 [file Table_1.DOC]

	
Varieties	Repeats	Average starch content	Standard Deviation	Standard Error	95% confidence level	Mini.
value	Maxi. value	
					Upper limit				
TX5	9	63.42178	.360453	.120151	63.14471	63.69885	62.552	63.789	
Liao138	9	67.33433	.482646	.160882	66.96334	67.70533	66.498	68.284	
By807	9	66.02322	2.237644	.745881	64.30322	67.74323	62.809	68.961	
GEMS18	9	63.59344	.513618	.171206	63.19864	63.98825	62.367	64.178	
GEMS29	9	66.94489	.463743	.154581	66.58842	67.30135	66.292	67.688	
CIMBL109	9	65.96511	.901511	.300504	65.27215	66.65807	64.994	67.617	
CML170	9	68.66200	.469068	.156356	68.30144	69.02256	67.899	69.619	
CIMBL2	9	65.68978	1.013215	.337738	64.91095	66.46860	64.419	66.971	
Shen137	9	67.69100	1.268530	.422843	66.71592	68.66608	65.790	69.267	
CIMBL77	9	68.71733	1.158098	.386033	67.82714	69.60753	66.938	70.290	
CIMBL146	9	68.40067	1.311704	.437235	67.39240	69.40893	66.939	70.304	
CIMBL17	9	68.82633	1.131654	.377218	67.95647	69.69620	67.030	69.932	
CIMBL56	9	68.25389	2.809133	.936378	66.09460	70.41318	64.548	71.800	
CML163	9	67.15456	.419622	.139874	66.83201	67.47711	66.292	67.668	
CIMBL154	9	66.19689	.562152	.187384	65.76478	66.62900	65.160	66.935	
CIMBL63	9	62.76378	.472621	.157540	62.40049	63.12707	62.238	63.674	
CML480	9	65.96189	.231150	.077050	65.78421	66.13957	65.656	66.488	
CIMBL22	9	68.42189	.963411	.321137	67.68135	69.16243	66.675	69.521	
CML118	9	67.71667	.938108	.312703	66.99557	68.43776	66.020	69.030	
CML223	9	69.15700	3.519226	1.173075	66.45188	71.86212	64.735	73.514	
Qi319	9	67.19967	.839095	.279698	66.55468	67.84465	65.732	68.162	
CIMBL52	9	65.02089	.659498	.219833	64.51395	65.52782	63.836	65.794	
CML479	9	69.57500	.444628	.148209	69.23323	69.91677	68.839	70.132	
CML122	9	67.10244	.906285	.302095	66.40581	67.79908	66.281	68.524	
CML31	9	63.77344	2.357253	.785751	61.96150	65.58539	60.775	67.030	
CML324	9	69.38078	1.742458	.580819	68.04141	70.72015	67.369	71.628	
CIMBL30	9	65.44122	1.313497	.437832	64.43158	66.45087	63.847	67.232	
CIMBL91	9	66.75644	2.202760	.734253	65.06325	68.44964	63.779	69.212	
CIMBL143	9	68.45856	1.420265	.473422	67.36684	69.55027	66.993	70.318	
CIMBL84	9	69.12611	1.095537	.365179	68.28401	69.96822	67.637	70.678	
CIMBL60	9	67.99233	.665866	.221955	67.48050	68.50416	66.970	68.953	
CIMBL112	9	65.56933	3.443490	1.147830	62.92243	68.21623	60.890	68.660	
CIMBL150	9	65.88911	1.090826	.363609	65.05063	66.72759	64.124	67.037	
CIMBL97	9	63.92611	.577907	.192636	63.48189	64.37033	62.709	64.631	
CIMBL67	9	66.72456	1.158913	.386304	65.83374	67.61538	65.213	68.187	
CIMBL1	9	68.79722	.605176	.201725	68.33204	69.26240	67.913	69.958	
CIMBL76	9	67.55644	1.269761	.423254	66.58042	68.53247	65.569	68.879	
CIMBL9	9	65.42133	.750708	.250236	64.84429	65.99838	64.241	66.302	
CML165	9	68.56644	1.350403	.450134	67.52843	69.60446	67.272	70.527	
CML470	9	63.99744	.939470	.313157	63.27530	64.71959	62.597	65.379	
CIMBL54	9	68.51233	.372099	.124033	68.22631	68.79835	68.006	69.394	
CIMBL80	9	66.62856	.858777	.286259	65.96844	67.28867	65.078	67.329	
CIMBL96	9	63.35011	.809513	.269838	62.72786	63.97236	62.260	64.370	
CIMBL127	9	63.33078	1.147263	.382421	62.44891	64.21264	61.793	64.881	
CIMBL136	9	68.92267	3.235562	1.078521	66.43559	71.40974	64.748	73.390	
CIMBL106	9	68.07778	2.248756	.749585	66.34923	69.80632	65.972	71.310	
CML304	9	67.78000	3.456517	1.152172	65.12309	70.43691	63.497	72.171	
CIMBL122	9	66.43522	2.951105	.983702	64.16680	68.70364	63.506	70.891	
CIMBL58	9	69.55322	2.707090	.902363	67.47237	71.63408	66.631	73.252	
CIMBL142	9	69.40189	3.301201	1.100400	66.86436	71.93942	64.935	72.520	
Yun46	9	66.28667	1.360358	.453453	65.24100	67.33233	64.283	67.555	
CIMBL117	9	69.94667	.802437	.267479	69.32986	70.56347	68.748	71.353	
CIMBL83	9	65.15256	2.374626	.791542	63.32726	66.97785	61.387	67.010	
CIMBL93	9	68.46689	.317680	.105893	68.22270	68.71108	67.808	68.937	
CIMBL48	9	70.45556	2.341200	.780400	68.65595	72.25516	67.088	73.138	
CIMBL59	9	66.24300	2.074021	.691340	64.64877	67.83723	63.332	68.924	
CIMBL92	9	69.75111	1.097853	.365951	68.90723	70.59500	67.384	70.844	
647	9	68.90267	.806547	.268849	68.28270	69.52263	67.434	70.071	
CML121	9	66.10111	.913530	.304510	65.39891	66.80331	64.511	67.040	
Nan21-3	9	66.26756	.495235	.165078	65.88688	66.64823	65.562	66.918	
CML162	9	66.50911	1.353991	.451330	65.46834	67.54988	64.331	68.688	
CIMBL72	9	65.86389	.889905	.296635	65.17985	66.54793	64.971	67.511	
CIMBL107	9	65.39600	1.063454	.354485	64.57856	66.21344	63.499	66.703	
CIMBL148	9	66.74644	.971389	.323796	65.99977	67.49312	65.395	68.100	
CIMBL10	9	66.36667	1.025748	.341916	65.57821	67.15513	64.636	67.583	
P138 	9	68.82022	1.411140	.470380	67.73552	69.90492	66.684	70.563	
CIMBL139	9	66.42922	1.640345	.546782	65.16834	67.69010	64.217	68.154	
CIMBL145	9	67.42344	1.467880	.489293	66.29513	68.55176	66.018	69.415	
CIMBL126	9	66.21189	.569574	.189858	65.77408	66.64970	65.312	66.957	
CIMBL102	9	67.74344	3.251383	1.083794	65.24421	70.24268	63.585	71.999	
CML139	9	66.64933	.952291	.317430	65.91734	67.38133	65.197	67.858	
CML168	9	67.05578	.314969	.104990	66.81367	67.29788	66.618	67.445	
CML113	9	68.62189	3.320973	1.106991	66.06916	71.17461	63.584	71.313	
CML426	9	62.48189	.669340	.223113	61.96739	62.99639	61.702	63.523	
CIMBL140	9	67.81089	1.920001	.640000	66.33505	69.28673	64.961	69.781	
CML338	9	66.42411	.943405	.314468	65.69895	67.14928	64.828	67.397	
CIMBL88	9	67.69978	1.452714	.484238	66.58312	68.81643	65.728	69.840	
CML423	9	66.51589	2.040794	.680265	64.94720	68.08458	64.118	69.179	
CIMBL153	9	66.43356	1.969506	.656502	64.91966	67.94745	63.362	68.225	
CIMBL71	9	66.86700	1.436290	.478763	65.76297	67.97103	65.736	68.795	
CIMBL144	9	68.33200	.750345	.250115	67.75523	68.90877	67.447	69.612	
CIMBL138	9	68.66333	.379593	.126531	68.37155	68.95511	68.230	69.389	
CIMBL50	9	69.49400	1.568796	.522932	68.28812	70.69988	67.629	71.729	
CIMBL4	9	67.02711	3.360972	1.120324	64.44364	69.61058	63.784	72.388	
CIMBL23	9	68.13222	.486546	.162182	67.75823	68.50621	67.363	68.915	
CIMBL105	9	64.26678	2.292679	.764226	62.50447	66.02909	62.363	67.873	
CIMBL49	9	70.50122	.419740	.139913	70.17858	70.82386	69.784	71.217	
CIMBL8	9	67.47356	3.054468	1.018156	65.12568	69.82143	65.113	71.931	
CIMBL98	9	63.04822	.667749	.222583	62.53494	63.56150	62.234	63.919	
CIMBL12	9	70.64456	.748695	.249565	70.06906	71.22005	69.276	71.739	
CIMBL61	9	65.36244	.666541	.222180	64.85010	65.87479	64.574	66.125	
CIMBL66	9	68.82967	.935631	.311877	68.11048	69.54886	67.473	69.906	
CIMBL87	9	67.56422	1.317812	.439271	66.55126	68.57718	65.752	69.016	
CIMBL157	9	67.42833	.627684	.209228	66.94585	67.91081	66.031	68.180	
CIMBL42	9	63.37133	1.821621	.607207	61.97111	64.77156	61.044	65.681	
CIMBL45	9	69.34356	.738726	.246242	68.77572	69.91139	68.500	70.860	
CML290	9	68.03700	1.641688	.547229	66.77509	69.29891	65.543	69.864	
CIMBL27	9	64.74178	.964008	.321336	64.00078	65.48278	62.789	65.731	
CIMBL21	9	67.07900	1.407352	.469117	65.99721	68.16079	64.618	68.841	
CML325	9	68.52822	1.031231	.343744	67.73555	69.32090	67.333	70.052	
CML473	9	68.81867	1.064651	.354884	68.00030	69.63703	67.593	70.331	
L3180 	9	67.23778	.719008	.239669	66.68510	67.79046	66.119	68.072	
Ye478	9	70.89289	2.491337	.830446	68.97788	72.80790	67.269	73.171	
TY3	9	69.30767	1.455287	.485096	68.18903	70.42630	67.971	71.327	
K22	9	66.66044	1.171312	.390437	65.76009	67.56079	65.281	68.310	
JH96C	9	68.10722	1.069720	.356573	67.28496	68.92948	66.675	69.655	
TT16	9	67.96600	.982323	.327441	67.21092	68.72108	67.086	69.722	
9782	9	66.80900	.120277	.040092	66.71655	66.90145	66.616	67.038	
Zheng653	9	67.93411	1.178501	.392834	67.02823	68.83999	65.778	69.467	
zheng58	9	65.51200	1.407803	.469268	64.42987	66.59413	63.886	67.790	
GEMS53	9	68.82889	2.023420	.674473	67.27355	70.38423	65.652	70.461	
ZZ03	9	61.32533	.507978	.169326	60.93487	61.71580	60.435	61.836	
Sy1035	9	69.70478	.188272	.062757	69.56006	69.84950	69.443	69.976	
Ji53	9	68.07411	.613411	.204470	67.60260	68.54562	67.084	68.781	
Ye52106	9	67.03200	.799632	.266544	66.41735	67.64665	66.128	68.172	
Wu109	9	68.78133	1.260913	.420304	67.81211	69.75056	67.401	71.312	
By843	9	66.32667	.677934	.225978	65.80556	66.84777	65.005	67.462	
GEMS25	9	69.45767	.454336	.151445	69.10843	69.80690	68.702	70.037	
Ry737	9	67.78000	.400288	.133429	67.47231	68.08769	67.217	68.331	
Sy3073	9	65.00800	.245701	.081900	64.81914	65.19686	64.700	65.384	
Sy1052	9	66.01567	1.155240	.385080	65.12767	66.90366	64.252	67.598	
7381	9	69.49267	.720502	.240167	68.93884	70.04649	68.426	70.727	
Qi205	9	68.39267	2.058958	.686319	66.81001	69.97532	65.071	70.225	
Liao5263	9	65.71167	.968708	.322903	64.96705	66.45628	64.162	67.360	
1323	9	66.45889	.398745	.132915	66.15239	66.76539	65.639	66.945	
ZZ01	9	67.24089	1.149827	.383276	66.35705	68.12472	66.226	69.657	
LXN	9	66.27556	.944371	.314790	65.54965	67.00146	65.132	67.848	
By4839	9	68.00933	1.044687	.348229	67.20632	68.81235	66.997	69.777	
U8112	9	61.66267	.878588	.292863	60.98732	62.33801	60.551	63.140	
4019	9	65.38378	.995167	.331722	64.61882	66.14873	64.118	66.599	
Ji853	9	64.16756	.461688	.153896	63.81267	64.52244	63.412	64.641	
Zhong69	9	68.08722	.919961	.306654	67.38008	68.79437	66.700	69.181	
GEMS30	9	62.75022	.857110	.285703	62.09139	63.40906	61.896	64.626	
7884-4Ht	9	64.36222	.268221	.089407	64.15605	64.56840	64.049	64.789	
Gy462	9	66.25100	1.338203	.446068	65.22237	67.27963	64.551	67.964	
chuan48-2	9	66.93933	2.651452	.883817	64.90125	68.97742	64.377	70.649	
GEMS14	9	68.30633	.539464	.179821	67.89166	68.72100	67.543	69.168	
GEMS17	9	68.08767	1.232680	.410893	67.14015	69.03519	66.700	69.910	
Liao159	9	67.11789	.788296	.262765	66.51195	67.72383	66.217	68.258	
Dan4245	9	64.52378	1.774597	.591532	63.15970	65.88785	61.708	66.815	
835b	9	66.47189	1.121952	.373984	65.60948	67.33430	64.529	67.884	
GEMS49	9	65.67956	.774318	.258106	65.08436	66.27475	64.190	66.555	
By809	9	68.48611	.366557	.122186	68.20435	68.76787	67.842	68.952	
Si444	9	65.24333	1.692010	.564003	63.94274	66.54393	62.734	67.711	
04K5702	9	67.10556	1.107108	.369036	66.25456	67.95655	65.442	68.930	
GEMS47	9	65.13244	.769218	.256406	64.54117	65.72372	63.935	66.025	
WMR	9	65.41622	.731231	.243744	64.85415	65.97830	64.337	66.708	
FCD0602	9	61.53900	1.663335	.554445	60.26045	62.81755	59.209	63.452	
04K5672	9	67.73122	1.378298	.459433	66.67177	68.79068	65.634	69.170	
Dan3130	9	63.47033	.783744	.261248	62.86789	64.07277	62.594	64.683	
9642	9	66.02889	1.300348	.433449	65.02935	67.02843	63.897	67.682	
Ye8001	9	67.43600	.933212	.311071	66.71867	68.15333	66.047	68.655	
Mo17	9	68.72222	2.243613	.747871	66.99763	70.44682	67.028	72.436	
TY6	9	67.11367	2.422427	.807476	65.25162	68.97571	63.886	69.779	
GEMS5	9	67.61389	.702473	.234158	67.07392	68.15386	66.381	68.461	
Dan340	9	61.33656	.319932	.106644	61.09063	61.58248	60.654	61.824	
Sy1039	9	67.44200	1.633594	.544531	66.18631	68.69769	65.498	69.674	
GEMS12	9	67.85311	1.432731	.477577	66.75182	68.95441	65.936	69.476	
B111	9	71.58267	.455292	.151764	71.23270	71.93264	70.783	72.271	
K10	9	66.34689	1.139826	.379942	65.47074	67.22304	64.564	67.511	
DSB	9	70.89489	1.603070	.534357	69.66266	72.12712	69.197	73.412	
DH3732	9	66.41033	.768074	.256025	65.81994	67.00073	64.893	67.426	
GEMS51	9	66.76522	1.332430	.444143	65.74103	67.78942	64.144	68.040	
Liao5114	9	67.44811	1.053319	.351106	66.63846	68.25776	65.759	69.100	
Z2018F	9	65.84322	1.733716	.577905	64.51057	67.17587	63.568	68.260	
BS16	9	66.77767	1.316706	.438902	65.76556	67.78978	65.007	68.575	
Dan598	9	63.38389	3.596032	1.198677	60.61973	66.14804	58.667	67.724	
By804	9	65.22656	.436591	.145530	64.89096	65.56215	64.743	65.978	
TY7	9	67.70756	1.859965	.619988	66.27786	69.13725	65.135	70.133	
Gy246	9	70.91389	1.663425	.554475	69.63527	72.19251	68.844	72.880	
975-12	9	70.42278	1.898561	.632854	68.96341	71.88214	67.820	72.802	
TY1	9	65.98067	.566108	.188703	65.54552	66.41582	65.052	66.618	
GEMS65	9	65.80122	.928248	.309416	65.08771	66.51474	64.429	67.081	
Dan360	9	66.91067	.778454	.259485	66.31229	67.50904	65.472	68.339	
MO113	9	64.93722	2.610305	.870102	62.93076	66.94368	61.651	68.215	
3411	9	64.37822	1.230114	.410038	63.43267	65.32377	62.641	65.899	
GEMS41	9	65.22056	.447144	.149048	64.87685	65.56426	64.676	66.083	
ES40	9	68.87222	2.135098	.711699	67.23104	70.51340	65.972	71.577	
J4112	9	68.44689	.759892	.253297	67.86278	69.03099	67.262	69.632	
CF3	9	65.68789	2.141582	.713861	64.04172	67.33405	62.531	68.875	
Gy220	9	66.65922	.574698	.191566	66.21747	67.10097	65.828	67.353	
GEMS52	9	66.34400	1.107408	.369136	65.49277	67.19523	64.664	67.450	
Gy923	9	66.27600	1.837644	.612548	64.86346	67.68854	63.956	68.628	
GY386B	9	66.36278	.652974	.217658	65.86086	66.86470	65.578	67.631	
GEMS55	9	64.10878	.847617	.282539	63.45724	64.76031	62.733	65.333	
GEMS31	9	62.81500	.615281	.205094	62.34205	63.28795	61.952	63.695	
Ry684	9	66.96300	1.265906	.421969	65.98994	67.93606	65.397	68.639	
526018	9	66.32867	.973308	.324436	65.58052	67.07682	64.700	67.333	
W138	9	65.38800	1.571977	.523992	64.17967	66.59633	63.436	67.359	
GEMS27	9	68.00000	.926248	.308749	67.28802	68.71198	66.650	68.917	
JH59	9	70.76478	1.618124	.539375	69.52098	72.00858	68.284	72.250	
TY5	9	63.42556	1.500742	.500247	62.27198	64.57913	61.896	65.831	
GEMS39	9	68.66822	.996064	.332021	67.90258	69.43386	67.619	70.198	
GEMS15	9	67.14478	1.803709	.601236	65.75832	68.53123	65.238	69.755	
Ji846	9	68.53589	1.258858	.419619	67.56825	69.50353	66.258	69.913	
R08	9	69.87500	.623464	.207821	69.39576	70.35424	69.086	70.670	
5213	9	67.71633	.915953	.305318	67.01227	68.42040	66.560	69.149	
Zong31	9	63.89489	2.265733	.755244	62.15329	65.63649	61.202	67.094	
150	9	66.15133	.479703	.159901	65.78260	66.52007	65.303	66.824	
GEMS4	9	65.92489	2.892637	.964212	63.70141	68.14837	61.567	68.789	
M153	9	66.46967	.462680	.154227	66.11402	66.82531	65.562	67.251	
TY8	9	66.20656	.569531	.189844	65.76877	66.64434	65.264	66.942	
GEMS54	9	66.58667	.733721	.244574	66.02268	67.15065	65.271	67.631	
GEMS23	9	69.95122	1.151480	.383827	69.06612	70.83633	68.409	71.326	
GEMS11	9	68.06900	.685991	.228664	67.54170	68.59630	67.306	69.184	
IRF314	9	68.89600	.675043	.225014	68.37712	69.41488	67.626	70.148	
BGY	9	70.38444	.982950	.327650	69.62888	71.14001	69.012	72.253	
TY2	9	68.88556	.772722	.257574	68.29159	69.47952	67.252	69.718	
Shen135	9	69.41978	2.498892	.832964	67.49896	71.34060	66.303	72.420	
Zheng35	9	68.16889	.328652	.109551	67.91626	68.42151	67.682	68.624	
TY4	9	67.78867	.789885	.263295	67.18151	68.39583	66.583	68.782	
GEMS48	9	70.69722	.618575	.206192	70.22174	71.17270	69.772	71.516	
LK11	9	65.84844	.478703	.159568	65.48048	66.21641	65.234	66.782	
Tie7922	9	60.59533	.300168	.100056	60.36460	60.82606	60.121	60.957	
Sy1077	9	68.67556	.297550	.099183	68.44684	68.90427	68.281	69.065	
384-2	9	67.06522	.317337	.105779	66.82130	67.30915	66.629	67.529	
Xi502	9	64.05411	.123416	.041139	63.95924	64.14898	63.867	64.193	
GEMS66	9	65.36933	.110419	.036806	65.28446	65.45421	65.219	65.583	
GEMS58	9	69.90744	.331358	.110453	69.65274	70.16215	69.535	70.590	
BZN	9	67.06644	.208236	.069412	66.90638	67.22651	66.759	67.273	
GEMS1	9	70.39322	.470865	.156955	70.03128	70.75516	69.868	71.189	
Sy999	9	65.50222	.352499	.117500	65.23127	65.77318	64.824	65.927	
By855	9	64.54233	.291815	.097272	64.31802	64.76664	64.151	64.852	
GEMS6	9	64.42211	.138038	.046013	64.31601	64.52822	64.159	64.605	
Si446	9	69.07244	.183008	.061003	68.93177	69.21312	68.841	69.329	
B113	9	66.15000	.411130	.137043	65.83398	66.46602	65.423	66.878	
Ji63	9	68.38144	.259111	.086370	68.18227	68.58061	68.139	68.827	
812	9	70.94478	.225870	.075290	70.77116	71.11840	70.638	71.282	
4F1	9	66.82800	.427184	.142395	66.49964	67.15636	66.094	67.420	
B151	9	70.14211	.333106	.111035	69.88606	70.39816	69.497	70.520	
B110	9	64.57400	.544193	.181398	64.15570	64.99230	63.473	65.273	
ZaC546	9	67.85589	.153013	.051004	67.73827	67.97350	67.572	68.042	
GEMS20	9	67.35500	.276697	.092232	67.14231	67.56769	66.933	67.920	
TY11	9	65.95778	.160663	.053554	65.83428	66.08127	65.734	66.250	
HB	9	65.54622	.214144	.071381	65.38162	65.71083	65.320	65.966	
81162	9	69.08700	.059445	.019815	69.04131	69.13269	68.952	69.146	
Lv28	9	71.32622	.160666	.053555	71.20272	71.44972	71.035	71.575	
Zheng29	9	70.23500	.220823	.073608	70.06526	70.40474	69.813	70.525	
Tian77	9	66.77522	.171630	.057210	66.64330	66.90715	66.500	66.925	
501	9	66.68233	.107861	.035954	66.59942	66.76524	66.520	66.837	
Si273	9	67.50844	1.016826	.338942	66.72684	68.29005	66.291	68.886	
B73	9	67.39856	.217911	.072637	67.23105	67.56606	67.042	67.748	
3H-2	9	66.59611	.237446	.079149	66.41359	66.77863	66.297	66.873	
Gy237	9	65.54833	.442368	.147456	65.20830	65.88837	64.634	66.098	
Ry732	9	64.22867	1.302377	.434126	63.22757	65.22976	62.421	66.225	
CML228	9	67.38722	.215176	.071725	67.22182	67.55262	67.088	67.729	
GEMS3	9	65.49511	.199600	.066533	65.34169	65.64854	65.130	65.831	
GEMS32	9	67.05511	.811874	.270625	66.43105	67.67917	66.048	68.198	
GEMS21	9	70.34889	1.433166	.477722	69.24726	71.45052	67.745	71.981	
CIMBL38	9	68.76211	.370395	.123465	68.47740	69.04682	68.283	69.321	
CML189	9	62.72144	.311457	.103819	62.48204	62.96085	62.076	63.238	
CIMBL86	9	66.39278	1.652192	.550731	65.12279	67.66276	64.522	70.461	
CML432	9	69.17344	.186975	.062325	69.02972	69.31717	68.873	69.416	
CIMBL85	9	65.39533	1.507987	.502662	64.23619	66.55447	63.341	67.657	
K12	9	64.51867	.321507	.107169	64.27153	64.76580	63.976	65.143	
JY01	9	69.77600	.329474	.109825	69.52274	70.02926	69.431	70.229	
GEMS44	9	67.23233	.188817	.062939	67.08720	67.37747	66.964	67.614	
Sy1032	9	69.19656	.390164	.130055	68.89665	69.49646	68.444	69.696	
By813	9	66.08611	.218265	.072755	65.91834	66.25388	65.667	66.378	
GEMS28	9	64.46800	.062775	.020925	64.41975	64.51625	64.386	64.582	
D047	9	69.85833	.354736	.118245	69.58566	70.13101	69.169	70.369	
Total	2367	66.99199	2.414063	.049619	66.89469	67.08930	58.667	73.514	
